# Inhibition of Effector Function but Not T Cell Activation and Increase in FoxP3 Expression in T Cells Differentiated in the Presence of PP14

**DOI:** 10.1371/journal.pone.0012868

**Published:** 2010-09-23

**Authors:** Zohar Ochanuna, Anat Geiger-Maor, Adi Dembinsky-Vaknin, Dimitrios Karussis, Mark L. Tykocinski, Jacob Rachmilewitz

**Affiliations:** 1 Goldyne Savad Institute of Gene Therapy, Hadassah-Hebrew University Medical Center, Jerusalem, Israel; 2 Department of Neurology, Hadassah-Hebrew University Medical Center, Jerusalem, Israel; 3 Jefferson Medical College, Thomas Jefferson University, Philadelphia, Pennsylvania, United States of America; New York University, United States of America

## Abstract

**Background:**

T-helper polarization of naïve T cells is determined by a complex mechanism that involves many factors, eventually leading to activation of Th1, Th2, or Th17 responses or alternatively the generation of regulatory T cells. Placental Protein 14 (PP14) is a 28 kDa glycoprotein highly secreted in early pregnancy that is able to desensitize T cell receptor (TCR) signaling and modulate T cell activation.

**Methodology/Principal Findings:**

Prolonged antigen-specific stimulation of T cells in the presence of PP14 resulted in an impaired secretion of IFN-γ, IL-5 and IL-17 upon restimulation, although the cells proliferated and expressed activation markers. Furthermore, the generation of regulatory CD4^+^CD25^high^Foxp3^+^ T cells was induced in the presence of PP14, in both antigen-specific as well as polyclonal stimulation. In accordance with previous reports, we found that the induction of FoxP3 expression by PP14 is accompanied by down regulation of the PI3K-mTOR signaling pathway.

**Conclusions/Significance:**

These data suggest that PP14 arrests T cells in a unique activated state that is not accompanied with the acquisition of effector function, together with promoting the generation of regulatory T cells. Taken together, our results may elucidate the role of PP14 in supporting immune tolerance in pregnancy by reducing T cell effector functions along with augmenting Treg differentiation.

## Introduction

PP14 is a glycoprotein that belongs to the lipocalin superfamily, of which most members are extracellular proteins that function in transporting small hydrophobic ligands [1]. PP14, also known as glycodelin, appears in various glycoforms: 1) follicular PP14 (Glycodelin F) that is expressed in the follicular fluid and inhibits sperm-oocyte binding; 2) seminal PP14 (Glycodelin S) that is expressed in the seminal fluid and maintains the uncapacitated state of the sperm; and 3) the amniotic glycoform (Glycodelin A) [2]. Amniotic PP14 is mainly synthesized in secretory endometrial glands and by gestational decidua where it is regulated by progesterone and it is the major secretory protein of human endometrial epithelial cells during the luteal phase and early pregnancy [3]. Maternal serum levels of PP14 increase during the second half of the luteal phase and peak around the onset of menses. PP14 concentrations rise rapidly in early pregnancy, with the highest concentrations found in the decidua between the 6th and 12th week of gestation. A correlation between low serum levels of PP14 and susceptible abortion has been recognized. This and other evidences suggest that high levels of PP14 may have an important role for establishment, maintenance, and progression of pregnancy and for early survival of the developing fetoplacental unit [4].

In addition to contraceptive and pro-angiogenic activities of amniotic PP14 [5], [6] several reports in the late 1980's pointed to PP14's immunoregulatory potential, with its capacities to inhibit: 1) T cell proliferation in allogeneic two-way mixed lymphocyte reactions; 2) mitogenic responsiveness of lymphocytes to the lectin PHA; 3) pro-inflammatory cytokine (IL-1 and IL-2) production by immune cells and 4) natural killer (NK) cell cytotoxicity [4], [7], [8]. More recent studies have demonstrated that PP14 can induce apoptosis in T cells [9], [10], [11].

In the present and previous studies we have used recombinant PP14·Fcγ_1_ produced in the human kidney 293 cells. It has been previously shown that recombinant PP14 produced in these cells has the same type of carbohydrate structures as that of the amniotic PP14 [12].

Key findings from our group pertaining to PP14's mode of action, suggest a novel paradigm for immunoinhibition wherein PP14 finely tunes, rather than inactivates, T cell responses. These studies demonstrated that PP14 mediates its anti-inflammatory activity by elevating T cell activation thresholds, thereby rendering T cells less sensitive to a given level of T cell receptor (TCR) stimulation [13]. Molecularly, PP14's inhibition depends on the presence of the surface tyrosine phosphatase CD45 [14] as well as upon its access to triggered TCR within antigen-presenting cell (APC):T cell contact sites, where it decreases the stability of TCR-induced phosphoproteins, hence explaining the TCR desensitization effect [13]. PP14 binds to T cell surfaces in a carbohydrate-dependent fashion – preferentially binding to sialylated N-acetyllactosamine. This seminal insight creates an intriguing link between PP14 and other immunoregulatory lectins (namely, galectin-1 and CD22) whose activity depends upon CD45 [15]. This carbohydrate binding profile correlates well with PP14's preferential co-capping of CD45RA isoform (presumably due to high degree of sialylation) and preferential inhibition of CD45RA^+^ (naïve) T cells [16].

This mechanistic insight into PP14's threshold modulation activity at the molecular level provided a useful framework for further exploring PP14's impact on T cell responses. Testing PP14's impact on early responses during polyclonal activation of bulk T cells, we have previously demonstrated that PP14 preferentially inhibits Th1 responses as compared with Th2 [17], in accordance with their respective known TCR signaling thresholds [18] and as expected for a threshold-centered mechanism [13].

Armed with these findings for bulk polyclonal activated T cells, in the present study we moved to longer-term assays of antigen-specific T cells, in order to further elucidate the immunological effects of PP14, particularly on Th subset differentiation.

## Results

### PP14·Fcγ1-treated MBP-specific T cells fail to develop into T effectors

Previous experiments in our lab so far dealt with PP14's impact on early responses during polyclonal activation of bulk T cells. Under these conditions we have demonstrated that PP14 preferentially inhibits Th1 responses as compared with Th2 [17]. In order to determine the consequence of PP14 treatment on Ag-specific T cell polarization over time and the way it may shape T helper response, we first used PBMC from healthy donors that were stimulated with myelin basic protein (MBP). MBP-reactive T cells from healthy donors are presumed not to be encountering their cognate CNS auto-antigens, and are most appropriate for studies of Th polarization [19]. MBP-specific T cells were generated in the presence or absence of PP14·Fcγ1, as described in *Materials and Methods*, and then restimulated with MBP, this time without PP14·Fcγ1 in order to characterize the developmental fate of the cells. Previous studies have established transforming growth factor-β (TGF-β) as a potent regulatory cytokine with diverse effects on T cell proliferation and differentiation [20]. In order to better appreciate PP14's impact on T cell activation and differentiation we compared its effects to that of TGF-β by also stimulating cells with MBP for 2 weeks in the presence of TGF-β followed by restimulation with MBP, this time in the absence of TGF-β.

**Figure 1:**
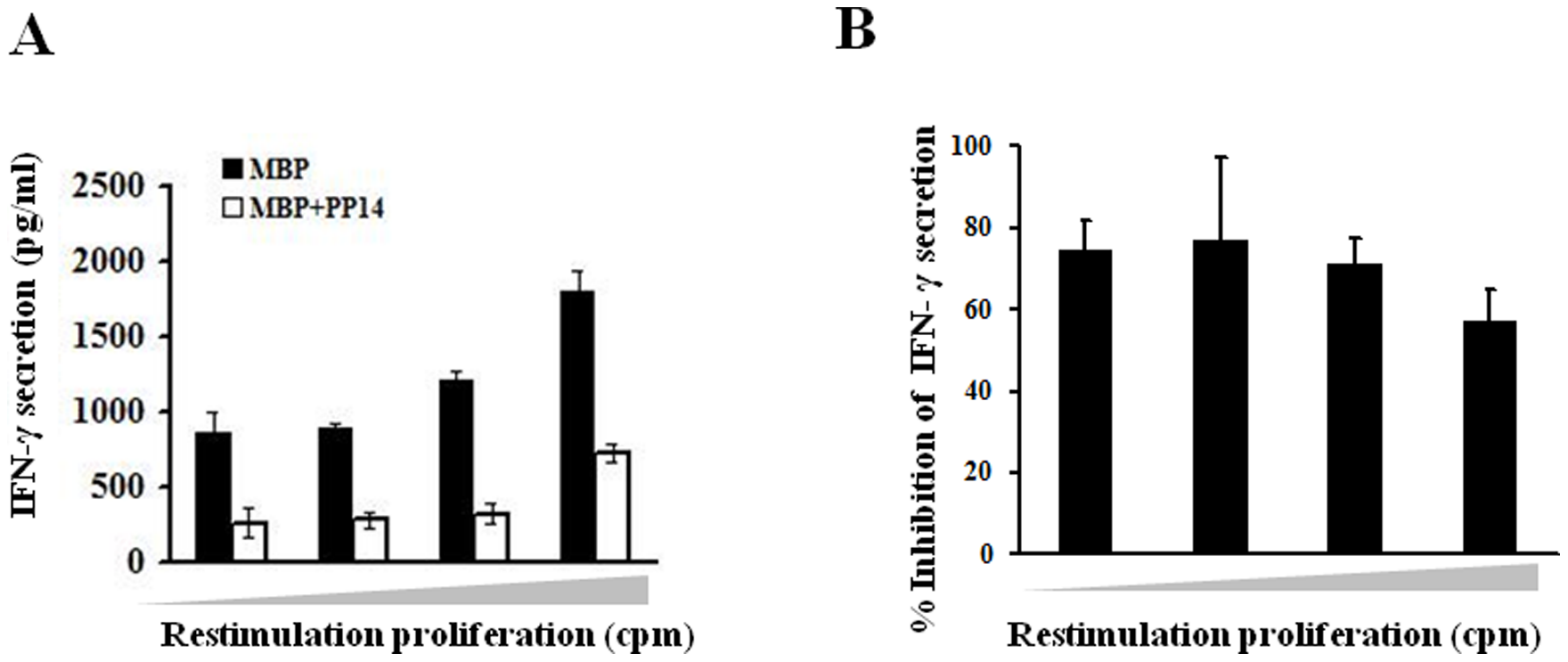
PP14·Fcγ1-treated cells secrete reduced levels of IFN-γ upon restimulation. PBMC from healthy donors were stimulated with MBP (20 µg/ml) in the presence or absence of PP14·Fcγ1 (50 µg/ml) for two weeks. After two weeks the cells were split and restimulated with MBP for three days. Conditioned media of responding cells were collected, from one plate, and pooled based on the proliferation measured by H^3^-thymidine incorporation in the parallel plate. Levels of secreted IFN-γ in conditioned media were measured by ELISA. *A*, One representative experiment out of 5 is shown. *B*, The average inhibition of IFN-γ secretion by PP14 in 5 experiments is shown.

Stimulation of the cells with MBP resulted in a predominant Th1 response, with IFN-γ being the main cytokine secreted with elevating levels that correlated with increasing proliferation. However, PBMC that were stimulated with MBP in the presence of PP14-γ during the first two weeks of stimulation showed an impaired IFN-γ response upon restimulation, at all levels of proliferative response (Fig. 1, A and B). Interestingly, there was no increase in Th2 or Th17 cytokine secretion (data not shown), indicating that the inhibition of Th1 response by PP14-γ is not accompanied by a skew toward these responses. Stimulation of the cells in the presence of TGF-β (during the first two weeks) led to complete inhibition of IFN-γ secretion upon restimulation (data not shown).

Previous reports suggested that patients with autoimmune diseases and specifically with multiple sclerosis (MS) have an immune deviation with predominance of Th17 response [21]. Cytokine secretion analysis in healthy donors versus MS-derived T cells revealed that while IFN-γ secretion in both groups was approximately the same, higher levels of IL-17 were secreted by MS-derived T cells as can be clearly seen when data presented as IFN-γ to IL-17 ratio. Interestingly, this difference in IFN-γ:IL-17 ratio was not only observed in an antigen-specific (MBP) stimulation but also in polyclonal stimulation with anti-CD3 (Fig. S1, A and B). Significantly, in both MBP and anti-CD3 stimulated MS-derived cells, PP14·Fcγ1 significantly inhibited both IFN-γ and IL-17 secretion (Fig. S1, C and D, respectively), suggesting it's capability to inhibit these cytokines in both normal and MS pathological conditions despite the predominant Th17 response in the latter.

### PP14·Fcγ1-treated MBP-specific T cells display an activated phenotype

**Figure 2:**
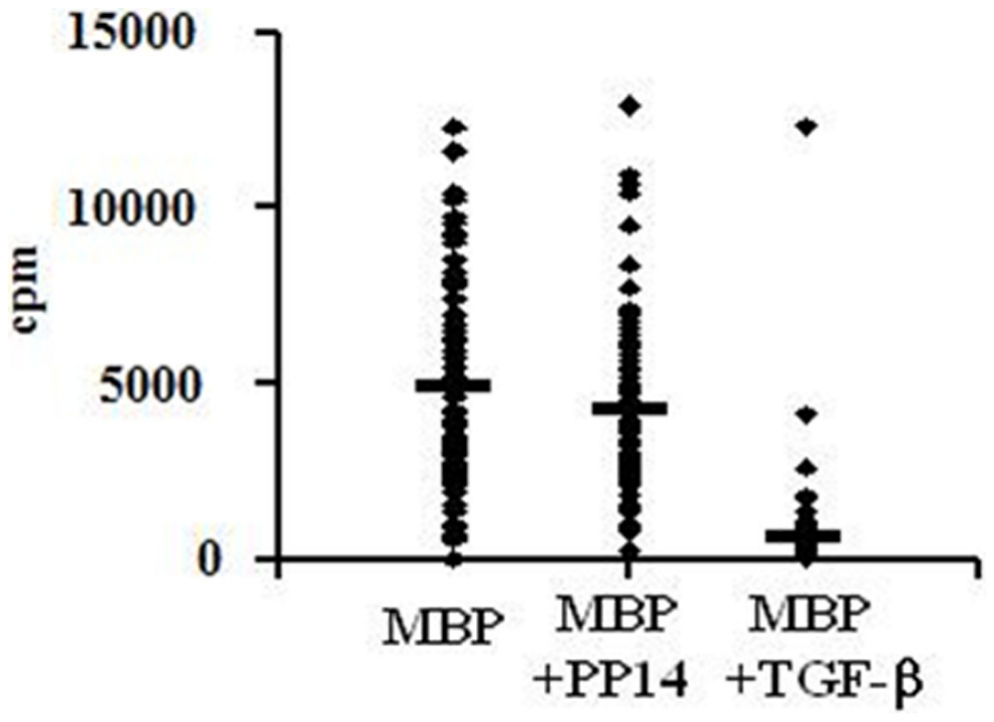
PP14·Fcγ1-treated cells proliferate as much as control MBP-stimulated cells upon restimulation. PBMC from healthy donors were stimulated with MBP (20 µg/ml) in the presence or absence of PP14·Fcγ1 (50 µg/ml) or TGF-β (5 ng/ml) for two weeks. After two weeks the cells were restimulated with MBP for three days and cell proliferation was tested by H^3^-thymidine incorporation (cpm). One representative experiment out of 5 is shown and each dot represents cell proliferation in a single well of a 96-well plate (p≤0.05).

Although PP14·Fcγ1 pre-treated cells exhibit impaired cytokine secretion, several observations indicate that these cells are activated. Despite their reduced levels of IFN-γ secretion (Fig. 1), PP14·Fcγ1 pre-treated cells proliferated as much as control MBP-activated cells upon restimulation (Fig. 2). In general, the proliferative response of PP14·Fcγ1 pre-treated cells was only slightly decreased as compared to control MBP-activated cells, as measured by their H^3^-thymidine incorporation. In contrast, activation of cells in the presence of TGF-β during the first two weeks resulted in complete inhibition of their proliferation upon restimulation (Fig. 2).

**Figure 3:**
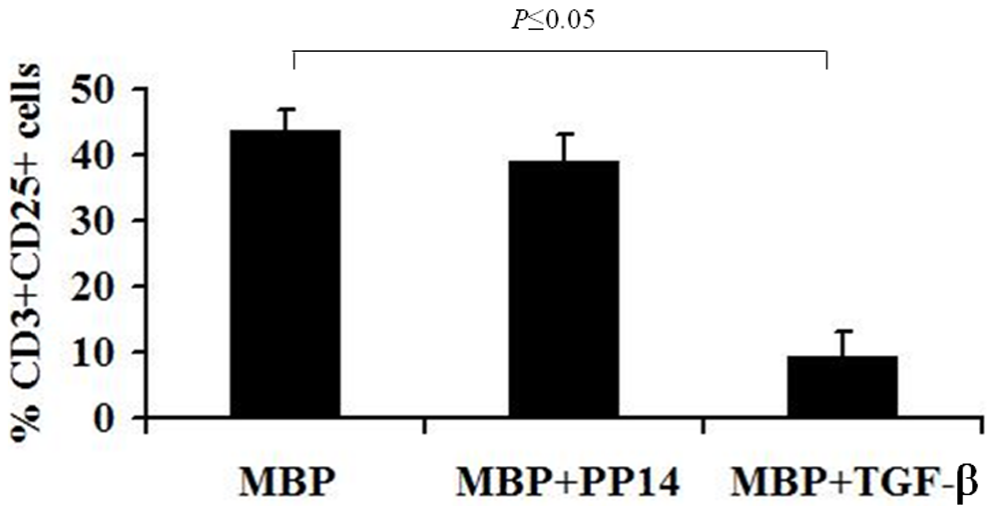
PP14·Fcγ1 pre-treated cells express the activation marker CD25. PBMC from healthy donors were stimulated with MBP (20 µg/ml) in the presence or absence of PP14·Fcγ1 (50 µg/ml) or TGF-β (5 ng/ml) for two weeks. After two weeks the cells were restimulated with MBP for three days and then were collected and immunostained for the expression of CD25 on T cells (CD3^+^ cells).

To further follow the activation status of the PP14-treated cells, T cells were immunostained for the expression of the activation marker, CD25. Flow cytometry analysis revealed that PP14·Fcγ1 pre-treatment did not significantly reduce the percentage of CD25^+^ cells, compared to control MBP-stimulated cells. In contrast, TGF-β significantly inhibited the expression of CD25 (Fig. 3). In addition, PP14·Fcγ1 pre-treated cells appeared to be activated similar to MBP-stimulated cells, as measured by lymphocyte blast transformation (Fig. S2, B and C, respectively). In contrast, TGF-β pre-treated cells are almost completely inhibited and display a scatter profile that is similar to that of non-stimulated cells (Fig. S2, D and A, respectively). Paralleling the respective effect of PP14·Fcγ1 and TGF-β on cell proliferation, these data further support the activated phenotype of PP14·Fcγ1-treated cells.

### Increased numbers of CD25^+^FOXP3^+^ T cells in PP14·Fcγ1 pre-treated cultures

**Figure 4:**
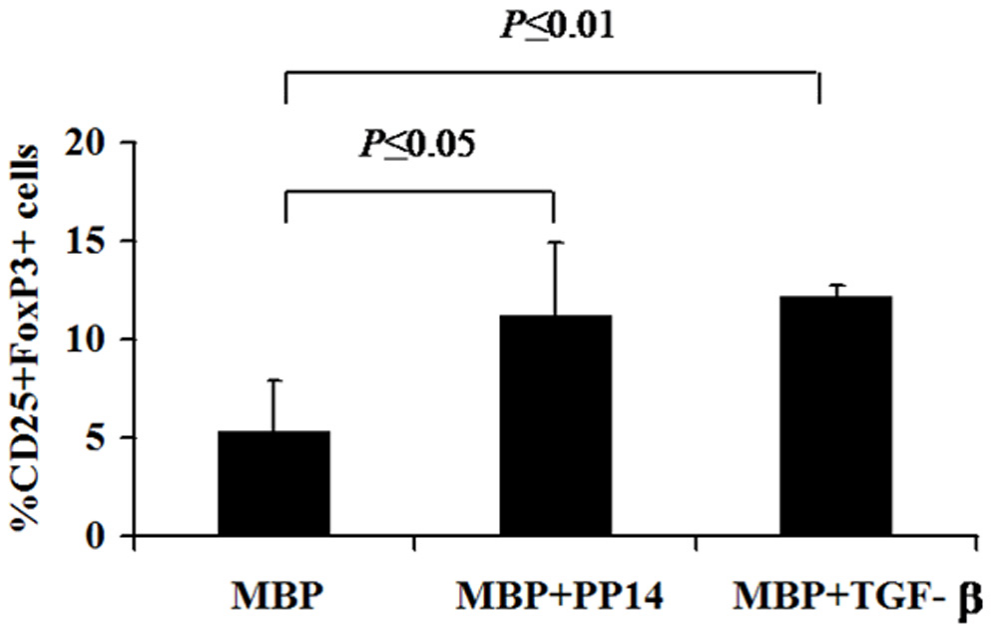
Increased numbers of CD25+FOXP3+ T cells in PP14·Fcγ1 pre-treated cultures. PBMC from healthy donors were stimulated with MBP (20 µg/ml) in the presence or absence of PP14·Fcγ1 (50 µg/ml) or TGF-β (5 ng/ml) for two weeks. After two weeks the cells were restimulated with MBP for three days and then were collected and immunostained for the expression of FoxP3 by activated CD25^+^ T cells.

Despite the activated phenotype of PP14·Fcγ1-treated cells, they do not seem to bear effector functions as demonstrated by their failure to secrete cytokines of either effector T cell lineage. This led to the hypothesis that PP14·Fcγ1 treatment might enhance a regulatory fate in the treated cells. Indeed, although the percentage of CD25^+^ cells in PP14·Fcγ1 pre-treated cells and control MBP-cells was similar, the percentage of FoxP3^+^ cells within these CD25^+^ cells was two-fold higher in PP14·Fcγ1 pre-treated cells as compared to control MBP-cells. TGF-β, the hallmark FoxP3 inducer, induced an average of 3-fold increase in the number of CD25^+^FoxP3^+^ cells among MBP-reactive activated cells (Fig. 4). These results were further corroborated at the level of mRNA using quantitative Real-Time PCR analysis with a two-fold and four-fold increase in FoxP3 gene expression over control MBP-activated cells, in PP14·Fcγ1 and TGF-β pre-treated cells, respectively (data not shown).

### PP14 induces *de-novo* generation of CD25^+^FoxP3^+^ cells

**Figure 5:**
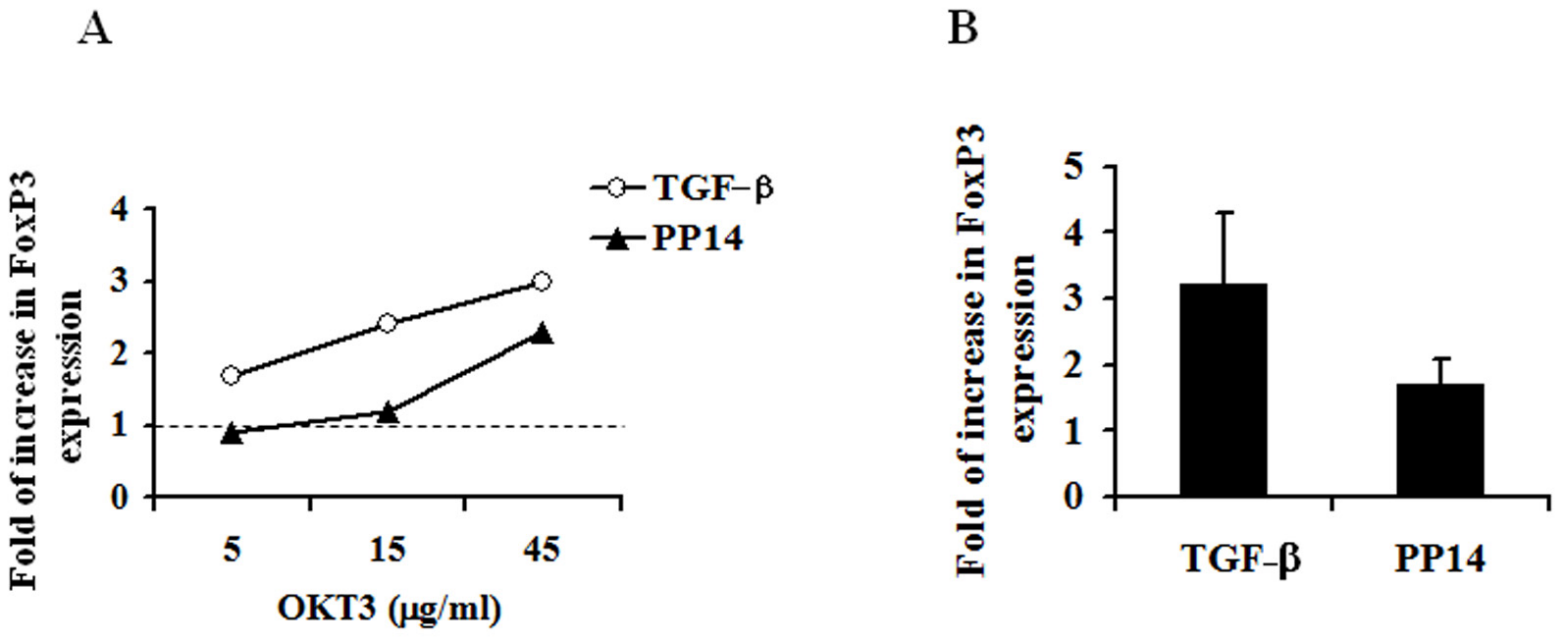
PP14·Fcγ1 induces *de-novo* generation of CD25+FoxP3+ cells. Naive CD4^+^CD25^−^ T cells were stimulated for one week with beads coated with various anti-CD3 concentrations in combination with soluble anti-CD28 (0.5 µg/ml) in the presence or absence of either PP14·Fcγ1 (50 µg/ml) or TGF-β (5 ng/ml). After one week the cells were collected and expression of CD25 and FoxP3 was analyzed using flow cytometry analysis. Results are presented as fold of increase in the percentage of cells expressing FoxP3 over that induced by TCR triggering alone (dotted line, *A*). One representative experiment is shown (*A*) and the average of seven separate experiments is shown in *B* (p≤0.05).

Since MBP-clones were generated from peripheral blood cells that already contain native peripheral CD25^+^FoxP3^+^ cells, their increased numbers in PP14·Fcγ1-treated wells can result either from expansion of the native Treg population or due to the emergence of adaptive FoxP3^+^ T cells from naïve cell population. To test this latter possibility we activated CD4^+^CD25^−^FoxP3^−^ T cells with various concentrations of immobilized anti-CD3 and anti-CD28 mAb with or without PP14·Fcγ1 or TGF-β, for 7 days. As previously reported with human cells [21], anti-CD3 stimulation by-itself induced FoxP3 expression in around 5 percent of the cells. Both PP14·Fcγ1 and TGF-β treatments increased the percentage of CD25^+^FoxP3^+^ T cells above that of anti-CD3 alone, although the effect of TGF-β was more pronounced (Fig. 5, A and B). Furthermore, PP14·Fcγ1-induced CD25^+^FoxP3^+^ cells are characterized as CD25^high^and were found to express the tumor necrosis factor (TNF)-superfamily member, GITR (glucocorticoid-induced TNF receptor family related protein), the typical marker of Treg [22], [23] (Fig. S3, B and D).

### The interplay of between PP14 and Retinoic Acid

**Figure 6:**
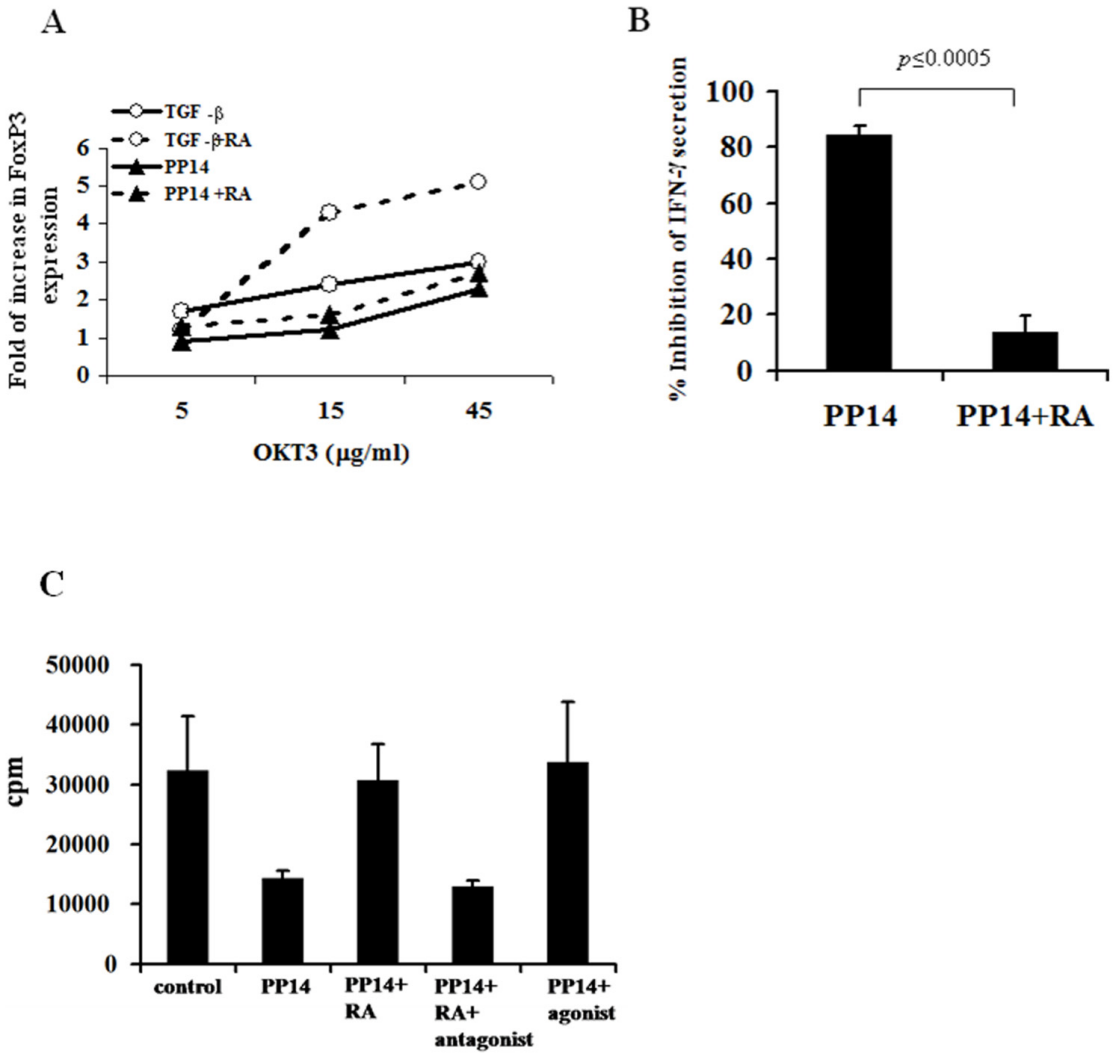
Retinoic acid enhances TGF-β but not PP14·Fcγ1-induced FoxP3 expression, and abrogates PP14·Fcγ1’s inhibitory activity. *A,* Naive CD4^+^CD25^−^ T cells were stimulated with beads coated with various anti-CD3 concentrations and anti-CD28 (0.5 µg/ml) for 1week in the presence or absence of either PP14·Fcγ1 (50 µg/ml) or TGF-β (5 ng/ml), with or without retinoic acid (10 nM). After one week the cells were collected and expression of CD25 and FoxP3 was analyzed using flow cytometry. One representative experiment out of 3 is shown. *B*, Conditioned media of stimulated cells was collected and analyzed for IFN-γ secretion using ELISA. Data is presented as percentage of inhibition of IFN-γ secretion by PP14. An average of three independent experiments is shown. *C*, Cells were stimulated with anti-CD3 (0.1 µng/ml) for three days in the presence or absence of g/ml) with or without 10 µnM of All-trans-retinoic acidµ1 (50γPP14•Fc agonist, α(RA). In the indicated wells, RA was replaced by the RAR antagonist GR-110 was added in αAM-580, whereas in other wells the RAR combination with RA. After three days conditioned media were collected and IFN-γ secretion was measured using ELISA. One representative experiment of three independent experiments is shown. The data represent the mean of triplicate samples (p≤.05).

Several recent studies have demonstrated that all-trans retinoic acid (RA) enhances the TGF-β–dependent FoxP3 expression and the conversion of naive T cells into Treg cells [24], [25], [26]. RA is of special interest here, given that PP14 is a lipocalin that can carry small hydrophobic ligands like retinoids in its hydrophobic pocket [27]. In an unpublished study we have previously shown that PP14 binds to two retinoids (all-*trans* retinoic acid and 13-*cis*-retinal) in a selective fashion, with no binding to two other retinoids (all-*trans*-retinal and all-*trans*-retinol). We therefore examined the effect of RA on PP14·Fcγ1-induced FoxP3 expression. The addition of RA to PP14·Fcγ1-treated cultures had a minimal effect on the percentage of PP14-induced CD25^+^FoxP3^+^ T cells while, as expected, enhanced TGF-β-induced FoxP3 expression (Fig. 6A). Interestingly, though RA had no effect on PP14·Fcγ1-induced FoxP3 expression, it significantly abrogated its T cell inhibitory activity as measured by IFN-γ secretion (Fig. 6B).

The addition of RA receptor-α (RARα) agonist could mimic RA effect on PP14's activity and the combination of RA with RARα antagonist abrogated RA's effect and restored PP14's inhibitory activity (Fig. 6C). These results suggest that RA's observed effect on PP14 activity is mediated through RA putative receptor and not as a result of its binding to PP14's hydrophobic pocket. In aggregate, these data indicate that in contrast to TGF-β, RARα signaling has no effect on PP14's induction of FoxP3 expression. Hence, we propose that PP14 and TGF-β induce FoxP3 expression via different pathways.

### Inhibition of PI3K mTOR signaling pathway by PP14

TGF-β is a potent inducer of FoxP3 expression, yet it has been suggested that in contrast to mouse, stimulation of human CD4^+^CD25^–^ T cells led to FoxP3 expression and acquisition of Treg activity via TCR signaling, independently of TGF-β signaling [21]. Specifically, Sauer et al have shown that that pre-mature termination of TCR triggering, either by TCR signal withdrawal or specifically by PI3K inhibitor significantly increased FoxP3 expression by naïve T cells, in the absence of TGF-β [28].

**Figure 7:**
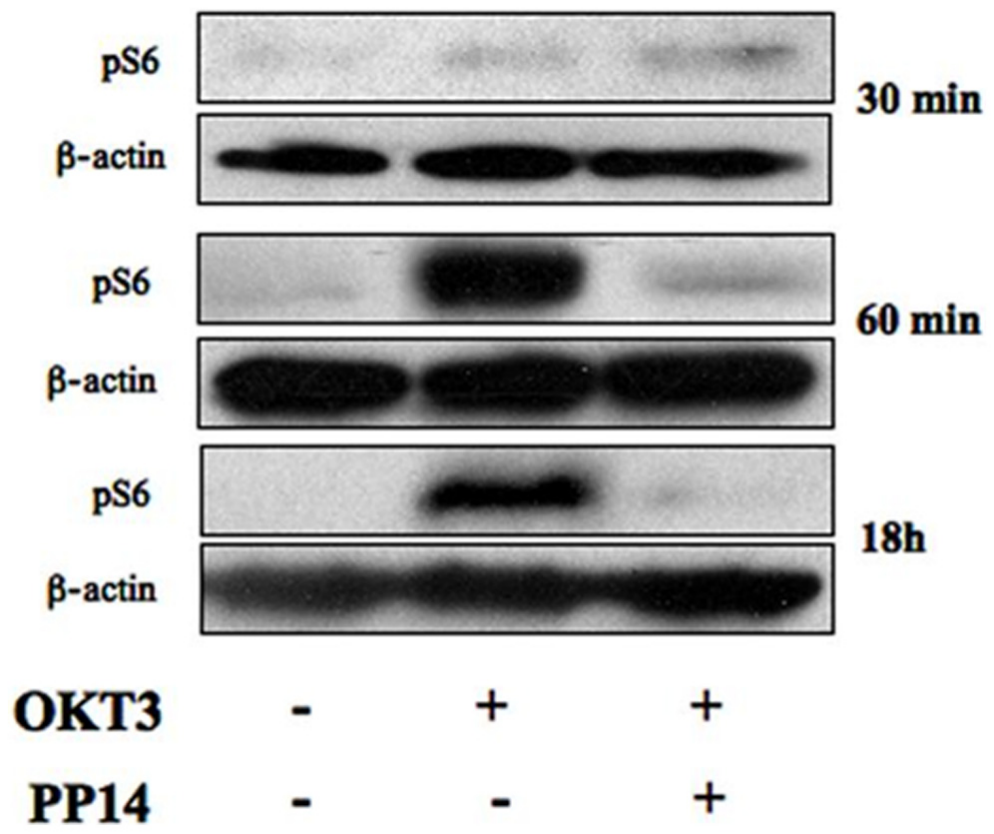
PP14·Fcγ1 inhibits the accumulation of TCR-induced phosphorylated ribosomal protein S6. Naive CD4^+^CD25^−^ T cells were stimulated with anti-CD3 (5 µg/ml) coated beads and anti-CD28 (0.5 µg/ml) in the presence or absence of PP14·Fcγ1. Cells were collected at the indicated time points after stimulation, lyzed and phosphorylated S6 was analyzed using Western blot analysis. Immunoblotting of actin reveals relative amounts of protein in each lane (lower panels).

This finding supports the role of TCR-induced PI3K-AKT-mTOR signaling pathway in regulating FoxP3 expression [28]. Therefore, it is possible that PP14 induces FoxP3 expression as a consequent of its ability to modulate TCR signaling events [13], [14], [29] and possibly reduce the duration of PI3K–mTOR signaling pathway. For that end, CD4^+^CD25^–^ T cells were stimulated with immobilized-anti-CD3 mAb and anti-CD28 with or without PP14·Fcγ1 and phosphorylated S6 (pS6), a direct ligand of the mTOR pathway, was detected at various time points after stimulation using immunoblotting. Low levels of phosphorylation were detected 15 and 30 minutes after stimulation that were increased in the presence of PP14·Fcγ1 (Fig. 7, upper panel). At later time points, from 1 h to 18 h following stimulation, S6 phosphorylation was strongly enhanced, yet significantly inhibited by PP14·Fcγ1 at these later time points (Fig. 7, lower panel). It seems that PP14 shortens the duration of TCR-induced mTOR signaling, hence providing a possible mechanism for the induction FoxP3 expression in these cells.

## Discussion

Following activation, human naïve CD4^+^ T cells can develop into a range of T helper lineages characterized by distinct cytokine profiles with IFN-γ and IL-4 being the signature cytokines of Th1 and Th2 cells, respectively, and IL-17 for the newly identified lineage, Th17 [30], [31], [32], [33], [34], [35].

In the present study we explored the possible impact of the pregnancy associated immunomodulatory lipocalin, PP14, on this CD4^+^ T cells developmental fate. Several previous studies uncovered the T cell immunoregulatory properties of PP14, and defined key aspects of its mode of action. The pivotal findings were that PP14 targets an early stage of CD4+ T cell activation [14], [29] via a unique immunoregulatory mechanism — “rheostatic” desensitization of T cell receptor signaling [13]. In delving into molecular and cellular underpinnings of this rheostatic regulatory mechanism, it was determined that PP14 constitutes a unique class of lipocalin/lectin immunoregulator [16] that may function via perturbation of immune synapses and consequent interference with summation of TCR-triggered signals [29], [36].

One intriguing implication of this mode of action that was demonstrated using polyclonal activated T cells is the preferential inhibition by PP14 of Th1, as opposed to Th2, responses [17]. This latter observation has prompted us to suggest that PP14 may skew the course of T helper polarization towards Th2. The focus of early studies in our lab was on early events in T cell activation. In going beyond the early TCR triggering process and short term T cell responses we now attempted to understand how PP14 helps shape the emergent T helper cell (Th) repertoire. Hence, in the present study we focused on long-term assays of MBP-specific T cells, in order to address how PP14 may impact T helper subset polarization per se. For that end, we stimulated cells with MBP in the absence or presence of PP14·Fcγ1 (or TGF-β as a control) for two weeks, a period that is sufficient for T helper polarization [37], [38]. To test their polarization fate we then re-stimulated the cells with MBP (this time in the absence of PP14·Fcγ1 or TGF-β) and cell proliferation, activation and cytokine secretion were analyzed. MBP stimulation led to a predominant Th1 response, with dominant secretion of IFN-γ. As expected, PP14 pre-treated cells displayed impaired IFN-γ secretion. However, in contrast to our initial assumption that PP14·Fcγ1 treatment would result in the inhibition of Th1 response that is accompanied by a skew towards a Th2 response, our findings unravel an unexpected outcome. Despite the inhibition of IFN-γ secretion in PP14·Fcγ1 -treated cells we found almost no secretion of IL-5, IL-4, suggesting that inhibition of Th1 response by PP14·Fcγ1 is not accompanied by a shift towards a Th2 response. A third and newly defined subset of T helper cells, that are distinct from Th1 and Th2 cells, is the Th17 subset of cells that secrete mainly IL-17. Interestingly, the inhibition of Th1 response by PP14 is neither accompanied by a shift towards a Th17 response, as demonstrated by cytokine secretion profile of the cells showing no increase of IL-17 secretion.

Th17 cells play a major role in the pathogenesis of several autoimmune diseases, including Multiple Sclerosis (MS) and it is assumed that there is a general immune deviation in MS patients towards Th17 response [39]. Therefore, we tested the effect of PP14 on T helper response in MS-derived PBMC. First, we analyzed cytokine secretion of MS patients and healthy donors and found that although IFN-γ secretion was predominant in both, IL-17 secretion was significantly increased in MS patients, as can be seen when the ratio of IFN-γ to IL-17 is presented. Interestingly, this skew toward IL-17 secretion is seen when cells were activated specifically by MBP as well as by polyclonal activation using anti-CD3. This finding suggest that the skew is not specific for the antigen (i.e. MBP) that is driving MS, per se, but is a more general predisposition of these patients T cells.

Yet, despite the predisposed immune deviation of MS-derived PBMC, stimulation of these cells in the presence of PP14·Fcγ1 led to a significant inhibition of cytokine secretion of all T helper lineages.

We initiated this study with the expectation, based on our previous studies, that T cell activation and proliferation would be reduced by PP14. However, in the present study we observed an unexpected outcome. Although cells initially stimulated with MBP in the presence of PP14·Fcγ1 displayed an impaired cytokine secretion upon restimulation, these cells appeared to be activated as demonstrated by their proliferative response. Activation of the cells was also manifested by lymphocyte blast transformation, and expression of the activation marker, CD25. In contrast, cells that were initially stimulated in the presence of TGF-β, did not display any of the above mentioned activation parameters and resembled unstimulated cells.

Together, these results suggest that PP14 uncouples T cell activation events (expression of activation markers and proliferation from cytokine secretion) that are otherwise linked. Since we have previously demonstrated that in contrast to other T cell inhibitors (such as cyclosporine A) PP14 functions by decreasing the stability of TCR-induced phosphoproteins and hence desensitisizing TCR signaling events [13], we assume that by selectively interfering with the signal cascade initiated by TCR triggering, PP14 eventually prevents the completion of the differentiation and polarization into either lineages of effector T cells and expression of the appropriate cytokines.

Several reports support the idea that various signaling pathways regulate different T cell responses and as a consequence these apparently linked responses can be dissociated and be regulated separately. Dong et al. have shown that c-Jun amino-terminal kinase (JNK)^−/−^ T cells produced IL-2 and proliferated, however, production of effector T-cell cytokines did require JNK. Thus, the authors concluded that JNK is necessary for T-cell differentiation but not for naive T-cell activation [40]. In another study, McClain et al., have shown that pregnant mice show a reduction in the incidence and clinical severity of EAE. This effect was associated with reduced levels of TNF-α as well as IL-17 expression with no increase in Th2 responses. However, in these mice T cell proliferation and expression of activation markers were unaffected [41]. Interestingly, we have recently observed a similar phenomenon where human T cells stimulated in the presence of allogeneic lymphocytes derived from decidual tissue expressed activation markers and proliferated but their cytokine response was impaired [42]. These two latter findings are specifically appealing since PP14 is a major secretory protein of human endometrial epithelial cells during pregnancy and its concentrations rise rapidly in early pregnancy [3], [43], [44]. Our results demonstrating selective inhibition of T cell effector functions in PP14·Fcγ1-treated cells may provide a possible link between PP14 and immunoregulation by this unique decidual lymphocyte population [42], as well as the general changes in T cell responses during pregnancy [45], [46], [47], [48].

Besides Th1, Th2 and Th17 effector cells, naïve CD4^+^ cells can also differentiate into regulatory T cells (Treg). Regulatory T cells expressing the transcription factor FoxP3 represent a pivotal subset of Tregs for dominant control of adaptive immune responses in the periphery [49], [50]. This formation of distinct lineages of effectors or regulatory T cells from naïve cells in response to Ag stimulation is a characteristic of the adaptive immune system. The outcome of T cell responses, immunity or tolerance is critically dependent on the balance between effectors and regulatory T cells. Having shown that PP14 inhibits the generation of effector T cells, we further asked whether it might push the cells towards a regulatory fate. Our data demonstrated an increase of up to two-fold in percentage of FoxP3-expressing cells in the presence of PP14·Fcγ1, compared to untreated cultures.

The increase in the number of FoxP3-expressing cells in the presence of PP14·Fcγ1 can be attributed to either the expansion of already-existing peripheral Treg or, alternatively, to *de-novo* generation of Treg cells from naïve population. We evaluated *de-novo* generation of Treg by stimulating CD4^+^CD25^−^ FoxP3^−^ T cells in the presence of PP14·Fcγ1. It has been shown that *in vitro* TCR stimulation of human CD4^+^CD25^−^ T cells in the absence of TGF-β induces FoxP3 expression and conversion to Treg cells [21]. This induction of FoxP3 by TCR stimulation is unique to human T cells but not to mice T cells where TGF-β is indispensable and TCR stimulation by itself is not sufficient for FoxP3 expression [49]. In agreement with these studies, our data suggest that TCR stimulation by itself induces FoxP3 expression independent of TGF-β, and as expected stimulation in the presence of TGF-β induced a four-fold increase in the number of FoxP3 expressing cells, compared to TCR stimulation alone. Interestingly, PP14·Fcγ1 induced a two-fold increase in the number of FoxP3 expressing T cells that are characterized as CD25^high^ and GITR^high^ cells. Together, these results support the assumption that PP14 can induce *de-novo* generation of Treg although not excluding the possibility that in the MBP-stimulated cultures expansion of native Treg cells takes place, as well.

TGF-β signals by binding its cell-surface serine/threonine kinase receptors, which in turn phosphorylate Smad2 and Smad3 that enter the nucleus and regulate FoxP3 gene expression [26], [51], [52]. Retinoic acid (RA) is known to strongly enhance the TGF-β-dependent conversion of naïve T cells into FoxP3^+^ T cells by increasing the expression and phosphorylation of Smad3 [26]. We asked whether it would have a similar beneficial effect on PP14's induction of FoxP3 expression. Surprisingly, we found that unlike its positive effect on TGF-β signaling and FoxP3 induction, RA had no effect on PP14·Fcγ1-induced FoxP3 expression. In contrast, RA significantly abrogated the inhibitory effect of PP14·Fcγ1 and restored cytokine secretion. Significantly, this latter negative effect is mediated by the binding of RA to its putative receptor and not by binding to PP14's hydrophobic pocket, as RA receptor agonists could replace RA, and RA receptor antagonists abrogated this negative effect of RA on PP14 activity.

Taken together, it is most likely that TGF-β and PP14 induce FoxP3 expression through distinct pathways that in the case of PP14 does not involve the Smad signaling. Since, as mentioned above, in humans, FoxP3 expression can be directly induced by TCR stimulation even in the absence of TGF-β, it is possible that the regulation of FoxP3 expression by PP14 is based on its ability to modulate TCR-evoked signaling. Our earlier studies have shed light on the way PP14 modulates T cell responses. First, we have demonstrated that PP14 acts through the tyrosine phosphatase receptor, CD45 [14]. Moreover, PP14 does not seem to inhibit tyrosine kinase activity *per se* but rather impedes TCR signaling via an alteration of the local balance between tyrosine kinases and phosphatases and consequently shortening the phosphorylated state of TCR-induced phosphoproteins.

Since commitment of T cells to cytokine production and proliferation requires sustained TCR signaling for up to several hours, PP14 may modulate T cell activation by reducing the duration of TCR signaling. Sauer et al. have shown that premature termination of TCR engagement 18 h after TCR triggering or, alternatively, selective inhibition of the PI3K/Akt/mTOR pathway induces FoxP3 expression and Treg-like gene expression profiles, in a TGF-β independent way [28].

In agreement with our previous observation [29], we demonstrated that S6 phosphorylation, an event regulated by mTOR [53], [54], [55], is not simply sustained but is gradually accumulating over time following TCR triggering. In accordance with the above proposition for PP14 activity we show that PP14·Fcγ1 inhibits S6 phosphorylation at late but not early time points. This may suggest that the observed increase in FoxP3 expression (and most likely the inhibition of effector functions) in PP14·Fcγ1-treated cells is a result of premature termination of TCR signaling and specifically of the PI3K/Akt/mTOR pathway. Interestingly, our results are strikingly supported by a recent study published by Powell et al. [56] showing that mTOR-deficient T cells display normal activation but fail to differentiate into Th1, Th2 and Th17 effector cells. The normal activation of the cells is attributed to the intact AKT activation upstream to mTOR. Moreover, under normal activating conditions, T cells lacking mTOR differentiated into Foxp3^+^ regulatory T cells in the absence of exogenous TGF-β. Specifically, it was shown that Treg differentiation in mTOR-defficient T cells is mediated by TORC2 signaling. Normally, TORC2 signaling is negatively regulated by TORC1, and indeed in this study TORC1 signaling was absent, as confirmed by impaired S6 phosphorylation.

Taken together, it appears that through regulating mTOR signaling PP14 may have an important influence on determining antigen-induced fate with regard to the development of effector versus regulatory T cells. Therefore, PP14 appears to have a potential therapeutic effect in T-cell mediated autoimmune diseases by preventing effector T cell development and promoting immune tolerance.

## Materials and Methods

### Ethics Statement

Blood samples from healthy donors were obtained from Hadassah Medical Center Blood Bank under approval of Hadassah Medical Center Helsinki Ethics Committee. Blood samples of MS patients were obtained from 14 non-treated patients (8 females, 6 males) with relapsing-remitting MS (EDSS 0.5–7) under approval of Hadassah Medical Center Helsinki Ethics Committee following signing on informed consent. The age range of the patients was 20–64 years with a mean age of 35 years.

### Cells

Peripheral blood mononuclear cells (PBMC) were purified from the venous blood of healthy donors or MS patients by density gradient centrifugation using Ficoll-Hystopaque (Sigma Aldrich, St Louis, MI, USA), as previously described [42]. The cells were cultured in RPMI medium (Biological Industries, Beit-Haemek, Israel) supplemented with 10% heat inactivated FCS, 2 mM glutamine and penicillin/streptomycin (Biological Industries) at 37°C, 5% CO2 incubator. CD4^+^CD25^−^ naïve T cells were purified by first isolating CD4^+^ T cells from the venous blood of healthy donors using RosetteSep human CD4 T cell enrichment mixture (StemCell Technologies, Vancouver, Canada). Next, CD25^+^ cells were depleted using anti-CD25 microbeads with magnetic cell isolation system (Miltenyi Biotec, Bergisch Gladbach, Germany). The resulting cell population was CD4^+^CD25^−^ and FoxP3^−^ cells as verified by flow cytometry analysis. CD4^+^CD25^−^ cells were cultured in 24-well plates (10^6^ cells/well) in RPMI 1640 medium (Biological Industries) supplemented with 10% heat-inactivated FCS, 2 mM glutamine, and penicillin/streptomycin (Biological Industries) at 37°C, 5% CO2 incubator.

### Production of PP14·Fcγ1

Stable 293 cell (ATCC) transfectants secreting PP14·Fcγ1 [14] were grown in DMEM medium, hygromycin B (200 µg/ml; Invitrogen), 10% heat inactivated FCS, 2 mM glutamine and penicillin/streptomycin (Biological Industries) at 37° C with 5% CO2. For protein purification, culture media of 293 cells secreting PP14·Fcγ1 was gradually replaced with serum-free DCCM-1 media (Biological Industries). After 48 hours of serum-free culture the conditioned media were collected and passed through a chromatographic column (C10/10, Amersham) prepared with Protein-A sepharose beads (Sigma-Aldrich, St. Louis, MO), at room temperature. Column was then washed with 20 mM Phosphate buffer pH 7 and 100 mM citrate pH 6. PP14-Fc γ1 was eluted from column with 100 mM citrate pH 3 and eluted fraction was titrated with 1M TRIS pH 9. PP14·Fcγ1 was concentrated and buffer exchanged into 40 mM citrate pH 6 using Centricon-10 filters (Millipore, Bradford, MA).

### Generation of MBP-specific T cells

For MBP activation experiments, PBMC from healthy donors or MS patients were plated in round bottom 96-well microplates (2×10^5^ cells/well), in the presence of 20 µg/ml of bovine myelin basic protein (MBP, Sigma-Aldrich) and in the absence or presence of either PP14·Fcγ1 (50 µg/ml) or TGF-β (5 ng/ml, R&D Systems, Minneapolis, MN). After two weeks, the cultures were split into two replicate plates that were restimulated with irradiated autologous PBMC (350 Rads, 10^5^ cells/well) and MBP, for 3 days. One of the replicate plates was pulsed with H^3^-thymidine (1 µCi/well, Amersham, Arlington Heights, IL) and wells exhibiting at least 3-fold higher H^3^-thymidine incorporation as compared to control wells were considered MBP-reactive wells. Cells and media from the corresponding wells in the replicate plate were collected either individually or as pools according to their proliferative response, and were further analyzed by flow cytometric analysis and ELISA, respectively.

### Induction of *de novo* expression of FoxP3

CD4^+^CD25^−^ cells were stimulated for one week with anti-CD3 mAb (OKT3; eBioscience) immobilized at the indicated concentrations on protein A-sepharose beads (Sigma-Aldrich) in combination with soluble anti-CD28 mAb (0.5 µg/ml; R&D Systems), in the presence or absence of PP14·Fcγ1 (50 µg/ml) or TGF-β (5 ng/ml, R&D Systems). In the indicated experiments the following treatments were added: 10 nM All-Trance Retinoic acid (RA; Sigma-Aldrich), 1 µM AM-580 RA-agonist or 5 µM GR-110 RA-antagonist (Biomol International). After one week the cells and conditioned media were collected and analyzed by flow cytometry and ELISA for the analysis of FoxP3 expression and cytokine secretion, respectively.

### Cytokine secretion analysis

PBMC were stimulated with MBP for two weeks and then restimulated with MBP as described in *Generation of MBP-specific T cells*. 72 hours after restimulation conditioned media were collected and IFN-γ, IL-5 and IL-17 levels were analyzed by ELISA (R&D Systems). Conditioned media were further analyzed using FlowCytomix human Th1/Th2 11plex KIT (Bender MedSystems, Burlingame, CA). In experiments using CD4^+^CD25^−^, cells were stimulated for one week, as described in *Induction of de novo expression of FoxP3*, and conditioned media were analyzed for IFN-γ and IL-17 secretion by ELISA (R&D Systems).

### Flow cytometry

MBP-reactive cells were collected after 72 hours of restimulation and CD4^+^CD25^−^ were collected after one week of stimulation and analyzed by flow-cytometry. Cell-surface markers were detected using the following Abs in various combinations: APC-conjugated mouse anti-human CD3 (IQ Products, Groningen, The Netherlands), FITC-conjugated mouse anti-human CD4 and PE-conjugated mouse anti-human CD25 (eBioscience). GITR expression was detected using APC-conjugated mouse anti-human GITR Ab (R&D Systems). FoxP3 expression was detected by intracellular staining using anti-human FoxP3 staining set with FITC-conjugated rat anti-human FoxP3 Ab (eBioscience), according to the manufacturer instructions.

### Western Blot

Naïve CD4^+^CD25^−^ cells were stimulated with immobilized anti-CD3 mAb in combination with soluble anti-CD28 mAb, as described in *Induction of de novo expression of FoxP3*, in the presence or absence of PP14·Fcγ1. Cells were collected at various time points after stimulation and lyzed as previously described [57]. Lysates were separated by electrophoresis on 10% SDS-PAGE gels and then transferred to PVDF membranes (Bio-Rad, Hercules, CA). The blots were probed with anti- phospho-Ribosomal Protein S6 antibody (R&D Systems) and HRP-conjugated goat anti-rabbit IgG (Jackson ImmunoResearch, West Grove, PA) as a secondary Ab. Membranes were processed with ECL Plus Western Blotting Detection System (Amersham-Pharmacia Biotech), and exposed to Chemiluminescence BioMax Light Film (Kodak-Industries, Cedex, France). Following stripping, the membranes were re-probed with anti-β-Actin mAb (Sigma-Aldrich) and goat anti-mouse IgG in DakoCytomation Envision^+^System-HRP labeled Polymer (DakoCytomation, Carpinteria, CA).

### Statistical analysis

The student paired two-tailed t test was used. Values of p≤ 0.05 were considered significant.

## Supporting Information

Figure S1 PBMC from MS patients exhibit increased IL-17 secretion and are inhibited by PP14•Fcγ1. PBMC from healthy donors (n = 10) or MS patients (n = 3) were stimulated with MBP for two weeks and then restimulated with MBP for three days, as described in materials and methods (A and C). In a parallel experiment, PBMC from healthy donors (n = 22) and MS patients (n = 14) were stimulated with anti-CD3 (1 ng/ml) in 96-well plate (105/well) for three days (B and D). The levels of IFN-γ and IL-17 in the conditioned media were analyzed using ELISA and is presented as the ratio between IFN-γ and IL-17 in each experiment (A and B). In one experiment PP14•Fcγ1 (50 µg/ml) was added to a parallel set of MS-derived cells that were stimulated by either MBP (C) or anti-CD3 (D) as described above, and the levels of IFN-γ and IL-17 in the conditioned media was analyzed by ELISA. The data represent the mean of triplicate samples.
(0.14 MB TIF)

Figure S2 Blast transformation of PP14•Fcγ1 pre-treated cells upon restimulation. PBMC from healthy donors were stimulated with MBP (20 µg/ml) in the presence or absence of PP14•Fcγ1 (50 µg/ml) or TGF-β (5 ng/ml) for two weeks. After two weeks the cells were restimulated with MBP for three days and then were collected. The activation of the cells, as demonstrated by blast transformation, was analyzed using flow cytometry analysis. A, unstimulated cells, B, MBP-activated cells, C, PP14•Fcγ1 treated and D, TGF-β treated T cells.
(0.13 MB TIF)

Figure S3 PP14•Fcγ1-induced FoxP3-expressing cells are CD25^high^ and express the Treg hallmark receptor, GITR. Naive CD4+CD25^−^ T cells were stimulated with anti-CD3 coated beads and anti-CD28 (0.5 µg/ml) for one week in the presence or absence of either TGF-β (5 ng/ml; A, C) or PP14•Fcγ1 (50 µg/ml; B, D). After one week the cells were collected and the levels of CD25 and GITR expression in FoxP3 positive (black line) and negative cells (grey) were analyzed using flow cytometry.
(8.73 MB TIF)
