# Supplementary figures and images for: Inhibition of Effector Function but Not T Cell Activation and Increase in FoxP3 Expression in T Cells Differentiated in the Presence of PP14
Source: PLoS One. 2010 Sep 23;5(9):e12868. doi: 10.1371/journal.pone.0012868 (PMC2944811; doi:10.1371/journal.pone.0012868)

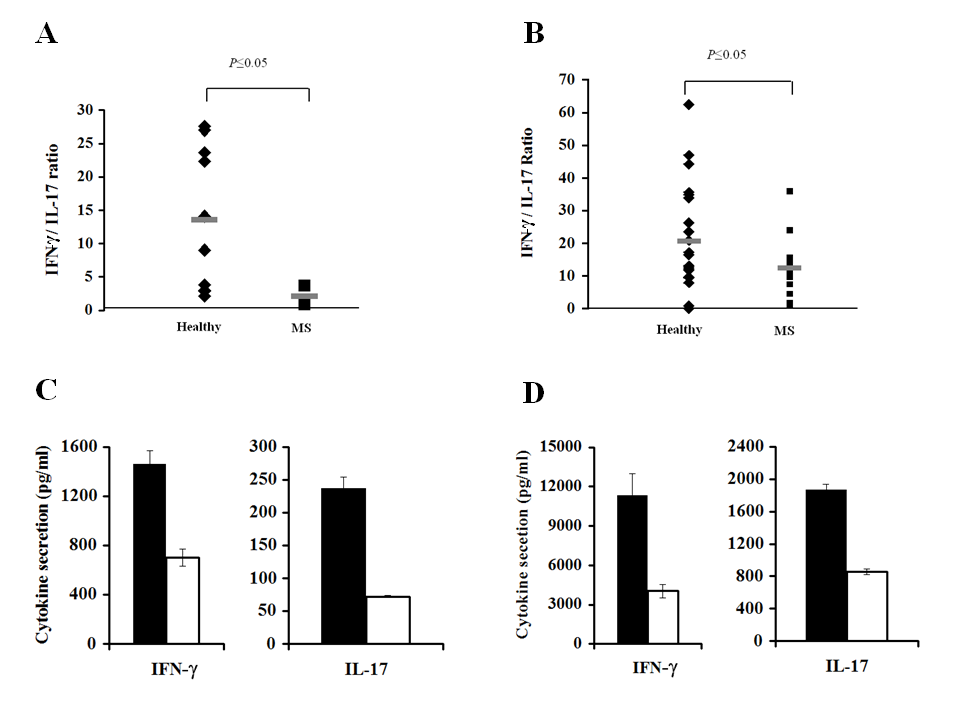

Supplement: Figure S1 — PBMC from MS patients exhibit increased IL-17 secretion and are inhibited by PP14•Fcγ1. PBMC from healthy donors (n = 10) or MS patients (n = 3) were stimulated with MBP for two weeks and then restimulated with MBP for three days, as described in materials and methods (A and C). In a parallel experiment, PBMC from healthy donors (n = 22) and MS patients (n = 14) were stimulated with anti-CD3 (1 ng/ml) in 96-well plate (105/well) for three days (B and D). The levels of IFN-γ and IL-17 in the conditioned media were analyzed using ELISA and is presented as the ratio between IFN-γ and IL-17 in each experiment (A and B). In one experiment PP14•Fcγ1 (50 µg/ml) was added to a parallel set of MS-derived cells that were stimulated by either MBP (C) or anti-CD3 (D) as described above, and the levels of IFN-γ and IL-17 in the conditioned media was analyzed by ELISA. The data represent the mean of triplicate samples. (0.14 MB TIF) [file pone.0012868.s001.tif]

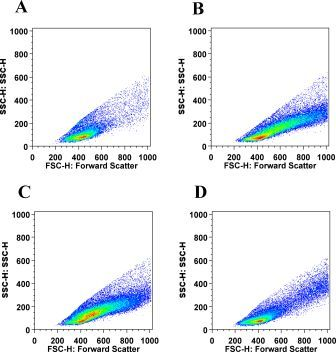

Supplement: Figure S2 — Blast transformation of PP14•Fcγ1 pre-treated cells upon restimulation. PBMC from healthy donors were stimulated with MBP (20 µg/ml) in the presence or absence of PP14•Fcγ1 (50 µg/ml) or TGF-β (5 ng/ml) for two weeks. After two weeks the cells were restimulated with MBP for three days and then were collected. The activation of the cells, as demonstrated by blast transformation, was analyzed using flow cytometry analysis. A, unstimulated cells, B, MBP-activated cells, C, PP14•Fcγ1 treated and D, TGF-β treated T cells. (0.13 MB TIF) [file pone.0012868.s002.tif]

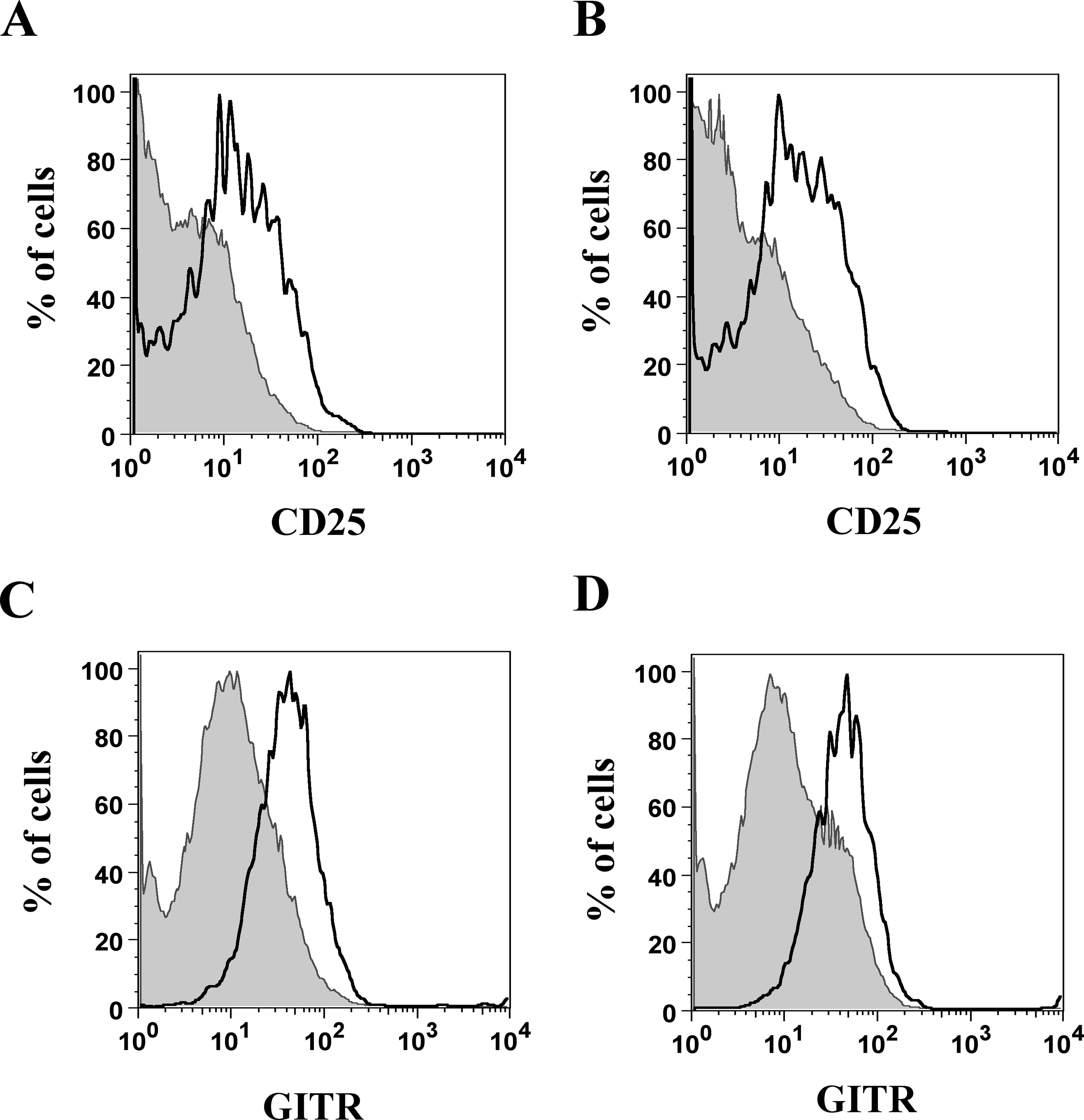

Supplement: Figure S3 — PP14•Fcγ1-induced FoxP3-expressing cells are CD25high and express the Treg hallmark receptor, GITR. Naive CD4+CD25− T cells were stimulated with anti-CD3 coated beads and anti-CD28 (0.5 µg/ml) for one week in the presence or absence of either TGF-β (5 ng/ml; A, C) or PP14•Fcγ1 (50 µg/ml; B, D). After one week the cells were collected and the levels of CD25 and GITR expression in FoxP3 positive (black line) and negative cells (grey) were analyzed using flow cytometry. (8.73 MB TIF) [file pone.0012868.s003.tif]
